# JQ1 Downregulates IL-20RA Expression in Triple Negative Breast Cancer Cells In Vitro and In Vivo

**DOI:** 10.3390/ijms27125233

**Published:** 2026-06-09

**Authors:** Valentina Maggisano, Salvatore Panza, Antonella Verrienti, Giovanni Enrico Lombardo, Stefania Catalano, Stefania Bulotta

**Affiliations:** 1 Department of Health Sciences, University “Magna Graecia” of Catanzaro, 88100 Catanzaro, Italy; vmaggisano@unicz.it; 2 Department of Experimental and Clinical Medicine, University “Magna Graecia” of Catanzaro, 88100 Catanzaro, Italy; salvatore.panza@unicz.it; 3 Department of Translational and Precision Medicine, Sapienza University of Rome, 00185 Rome, Italy; antonella.verrienti@uniroma1.it; 4 Department of Medicine and Surgery, University of Enna “Kore”, 94100 Enna, Italy; giovannienrico.lombardo@unikore.it; 5 Department of Pharmacy, Health and Nutritional Sciences, University of Calabria, 87036 Arcavacata di Rende (Cosenza), Italy; stefania.catalano@unical.it; 6 Centro Sanitario, University of Calabria, 87036 Arcavacata di Rende (Cosenza), Italy

**Keywords:** TNBC, TME, IL-20RA, BET-inhibitor, JQ1

## Abstract

The dynamic crosstalk between the tumor microenvironment (TME) and triple negative breast cancer (TNBC) cells plays a critical role in tumor progression and treatment resistance. Recent studies have highlighted the involvement of IL-20 receptor subunit alpha (IL-20RA) signaling in BC, where its overexpression modulates oncogenic pathways contributing to invasion and metastasis. Epigenetic dysregulation by Bromodomain and Extra-Terminal domain (BET) proteins critically influences key oncogenic pathways and cytokine expression in TNBC. Given that the BET-inhibitor JQ1 blocks TNBC cell growth, in this study we investigated its potential regulatory effects on the IL-20RA pathway. IL-20RA was found expressed across multiple BC cell lines compared to non-tumorigenic cells, with the highest levels detected in MDA-MB-231 and MDA-MB-468 cells. In both cell lines, JQ1 treatment significantly downregulated IL-20RA expression at gene and protein levels, accompanied by a reduction in the oncogenic JAK/STAT signaling pathway, and programmed death-ligand 1 (PD-L1) expression. Parallel in vivo experiments using TNBC xenograft models confirmed these findings, showing reduced IL-20RA and PD-L1 expression alongside decreased phosphorylation of JAK and STAT3. Overall, this study uncovers a novel interplay between BET inhibition and the IL-20RA/STAT3 axis, suggesting JQ1 as a valid therapeutic option for TNBC characterized by high IL-20RA expression.

## 1. Introduction

Breast cancer (BC) is one of the most common malignancies worldwide and represents a leading cause of morbidity and mortality among women [1,2]. Marked by extensive heterogeneity, BC comprises diverse biological subtypes with unique clinical behaviors and distinct therapeutic responses [3,4]. The most aggressive of these subtypes is triple-negative BC (TNBC), which lacks established therapeutic targets, namely the estrogen receptor (ER), progesterone receptor (PR), and human epidermal growth factor receptor 2 (HER2). This lack of targets, combined with multiple molecular and cellular alterations, endows TNBC cells with enhanced capacities for rapid proliferation and tissue invasion [5]. Currently, the primary treatment strategy for TNBC consists of surgery followed by cytotoxic chemotherapy, which remains the main non-surgical systemic approach [6]. The absence of targetable molecules constitutes a significant challenge in TNBC clinical management and underscores the need to identify novel prognostic factors and develop innovative therapeutic strategies [7,8,9].

Epigenetic-targeted antitumor therapies are currently demonstrating significant potential in clinical trials, utilized either as monotherapies or in combination regimens [10,11]. Bromodomain and extraterminal domain (BET) proteins function as epigenetic ‘readers’ that recognize and bind acetylated lysine residues on histone tails, a process that facilitates the recruitment of transcriptional machinery to drive the expression of oncogenes critical to various phases of tumor progression [12]. BET inhibitors (BETi) by preventing BET protein activity repress the expression of genes associated with cell proliferation and carcinogenesis [13,14,15]. JQ1 is one of the first BETi tested for its anticancer effects in both solid and hematological preclinical models of malignancies, with a selective activity against BRD4 that plays a crucial role in the survival of TNBC cells [16,17]. However, since drug resistance significantly limits its clinical use [18,19] emerging strategies including JQ1-based combinations, dual-targeting compounds, and novel proteolysis-targeting chimeras (PROTACs) designed for BET protein degradation have demonstrated promising efficacy in TNBC treatment [20,21,22]. The response of BC cells to therapies is critically influenced by the tumor microenvironment (TME) [23,24,25], a complex and heterogeneous system surrounding cancer cells composed of infiltrating immune cells, stromal cells, mesenchymal cells, cancer cells themselves, adipocytes, non-cellular tissue components and numerous cytokines [26]. Interleukins (ILs) are key components of the TME cytokine network and play a crucial role in promoting cancer progression. Among them, the interleukin-20 receptor subunit alpha (IL-20RA) has recently emerged as a relevant player in several cancers, including BC, where its overexpression correlates with poor prognosis and affects key tumor-intrinsic and microenvironmental features, such as proliferation, cell death and invasiveness [27,28]. Upon ligand binding, Il-20RA induces JAK activation and subsequent STAT phosphorylation that, translocating into the nucleus, modulates the transcription of genes implicated in cancer progression [29]. Therefore, IL-20RA may represent an attractive molecular target for the management of TNBC. Identification of JQ1-regulated target genes may provide new insights and a valid strategy to improve their therapeutic efficacy. Recently, it has been reported that BET inhibition by JQ1 modulates IL-20RA expression in colorectal cancer, but no data are currently available on the regulation of IL-20RA in BC [30]. In the present study, we investigated the ability of JQ1 to modulate IL-20RA expression and its downstream signaling pathway in BC cell lines in vitro, as well as in vivo by using tumor xenograft tissues collected from our previous work [31].

## 2. Results

### 2.1. Expression Levels of IL-20RA in Breast Non-Tumorigenic and Cancer Cells

**Figure 1:**
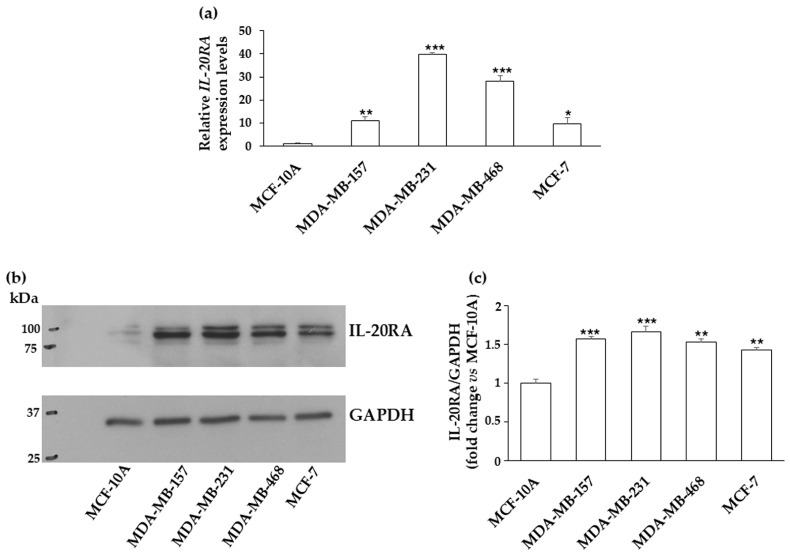
IL-20RA gene and protein expression in non-tumorigenic and malignant human breast cell lines. (**a**): IL-20RA mRNA levels in non-tumorigenic cell line MCF-10A and breast cancer cell lines. Relative gene expression is reported as means ± SD normalized to values in MCF-10A cells (equal to 1). (**b**,**c**): Representative Western blot images (**b**) and densitometric quantification (**c**) of IL-20RA expression in MCF-10A and breast cancer cell lines. GAPDH was used as loading control. Values are expressed as a ratio over MCF-10A equal to 1. *n* = 3. *p*-values were obtained by using the Tukey–Kramer multiple comparison test: * *p* < 0.05, ** *p* < 0.01, *** *p* < 0.001.

To confirm the involvement of IL-20RA signaling in BC, we investigate its gene and protein expression in human the non-tumorigenic breast epithelial cell line MCF-10A and several BC cell lines such as MDA-MB-157, MDA-MB-231, MDA-MB-468 and MCF-7, observing high levels across all tumor-derived cohorts with the highest expression detected in MDA-MB-231 and MDA-MB-468 cells as shown in Figure 1.

### 2.2. JQ1 Downregulates IL-20RA and Affects JAK/STAT Signaling Levels in TNBC Cells

**Figure 2:**
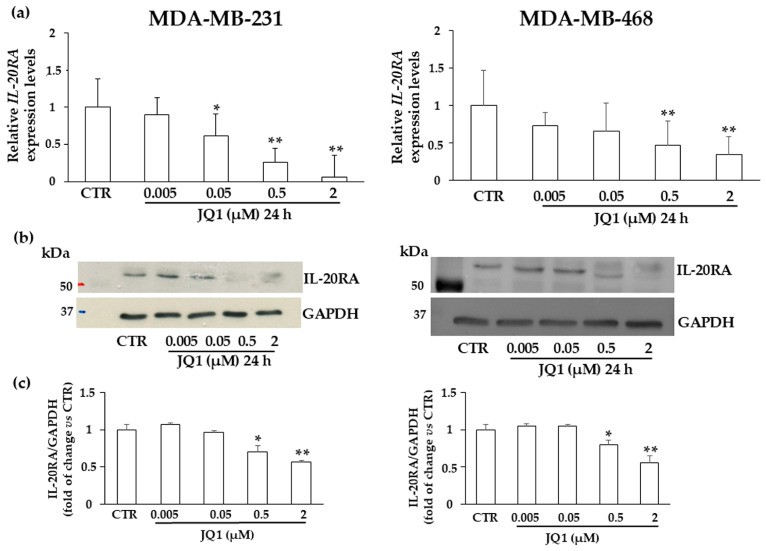
Effect of JQ1 on IL-20RA expression in MDA-MB-231 and MDA-MB-468 cells. (**a**): IL-20RA mRNA expression in TNBC cells after 24 h of treatment with JQ1. Relative gene expression levels are reported as the mean expression value normalized to the mean expression of the untreated cells indicated as CTR, (equal to 1). (**b**,**c**): Representative Western blot images (**b**) and densitometric quantification (**c**) of IL-20RA in TNBC cells exposed to JQ1. Data are normalized to GAPDH levels. *n* = 3. *p*-values were obtained by using one-way ANOVA with Tukey’s multiple comparison test: * *p* < 0.05, ** *p* < 0.01.

We have previously demonstrated that JQ1 significantly decreases cell viability in vitro and reduces neoplasm growth in vivo in TNBC models [31]. Against this background, to study the interplay between JQ1 and IL-20RA, we exposed MDA-MB-231 and MDA-MB-468 TNBC cells to various concentrations of JQ1 (0.005, 0.05, 0.5, and 2 µM) for 24 h. In both cell lines, treatment with JQ1 at 0.5 and 2 µM produced significant decreases in IL-20RA mRNA levels compared with non-treated cells (CTR) (Figure 2a). In line with these transcriptomic changes, protein levels were also found to be significantly reduced upon JQ1 treatment (Figure 2b).

**Figure 3:**
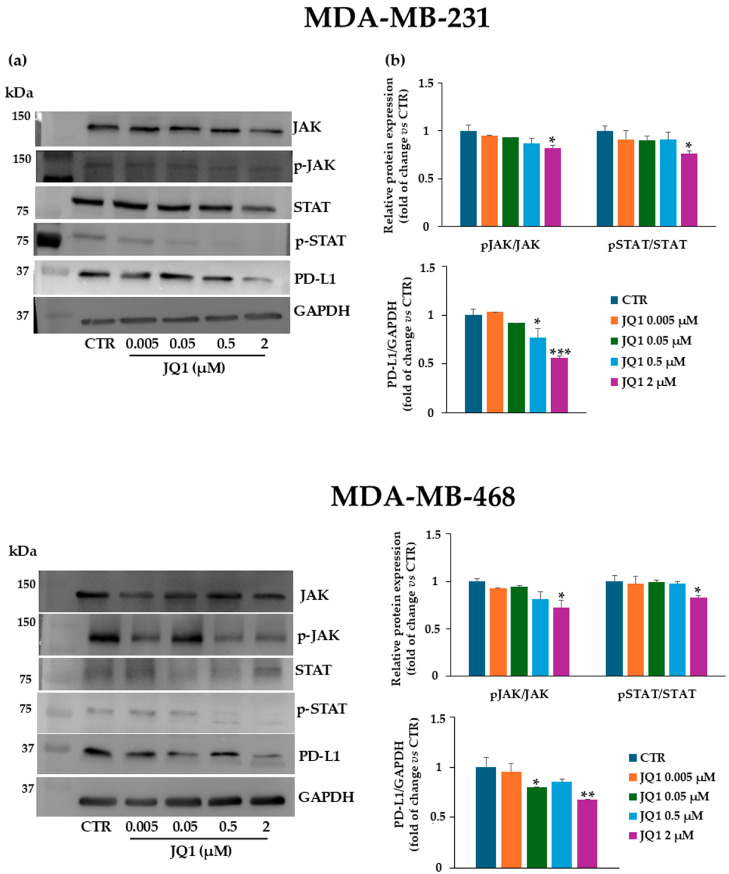
Effect of JQ1 on JAK/STAT signaling pathway and PD-L1 expression in MDA-MB-231 and MDA-MB-468 cells. (**a**): Immunoblotting of phosphorylated JAK (pJAK) and phosphorylated STAT (p-STAT), total form of the enzymes (JAK, STAT) and PD-L1 in MDA-MB-231 and MDA-MB-468 cells exposed to JQ1. GAPDH was used as loading control. (**b**): Densitometric analysis of pJAK/JAK, pSTAT/STAT and PD-L1. Data are expressed as fold over untreated cells indicated as control (CTR). *n* = 3. *p*-values were obtained by using one-way ANOVA with Tukey’s multiple comparison test: * *p* < 0.05, ** *p* < 0.01, *** *p* < 0.001.

In the same experimental conditions, to evaluate the involvement of the downstream signal transduction pathways activated by IL-20RA, we performed immunoblot analysis to measure the expression levels of phosphorylated STAT3, marker of activation of JAK/STAT pathway. As shown in Figure 3, we observed a decrease in phospho-STAT3 protein expression levels in JQ1-treated cells compared to the untreated cells. Moreover, since IL-20RA modulates programmed death-ligand 1 (PD-L1) expression in BC cells [27], we investigated its protein levels in both JQ1-treated cell lines, observing a significant downregulation (Figure 3).

### 2.3. JQ1 Affects IL-20RA Expression and JAK/STAT Signaling Levels in a BC Xenograft Model

**Figure 4:**
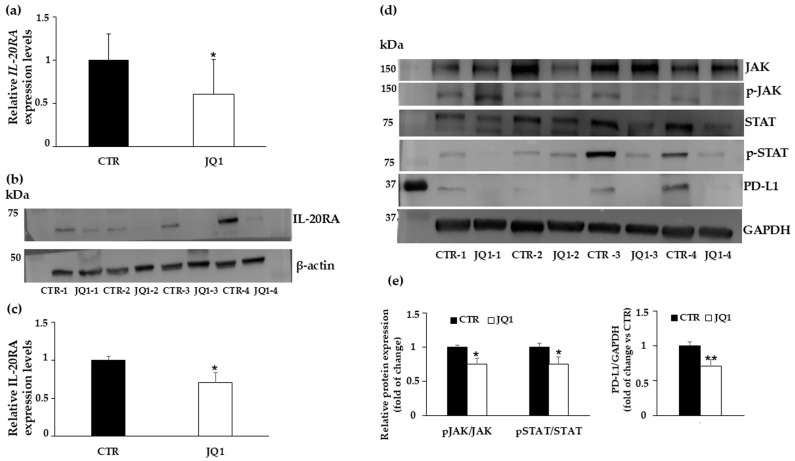
Effect of JQ1 on IL-20RA expression, JAK/STAT signaling pathway and PD-L1 expression in xenograft tumor tissues. (**a**): IL-20RA gene expression in xenograft tumor tissues treated (JQ1) or untreated (CTR) with JQ1. Relative expression levels are reported as means ± SD normalized to values in CTR (equal to 1). (**b**,**d**): Western blot analysis of IL-20RA (**b**); total and phosphorylated forms of JAK and STAT (pJAK, JAK, pSTAT, STAT) and PD-L1 protein levels (**d**). Lanes show protein extracts from individual tumors derived from four separate mice (numbered 1 to 4) per experimental group (CTR and JQ1-treated). (**c**,**e**): Densitometric quantification of the protein bands shown in (**b**,**d**), respectively. Values are set as mean ± SD (*n*= 4 mice per group) and normalized to CTR values (equal to 1). *n* = 3. *p* values were obtained with T-test with Welch’s correction: * *p* <0.05, ** *p* < 0.01.

To validate these findings in vivo, we employed the MDA-MB-231 xenograft model previously established in our laboratory [31]. Consistent with our in vitro data, treatment with JQ1 (20 mg/kg) attenuated IL-20RA gene and protein expression (Figure 4a–c) and impaired the JAK/STAT signaling pathway, as evidenced by decreased phospho-STAT3 levels compared to controls (Figure 4d,e). Furthermore, a significant downregulation of PD-L1 expression was observed in the tumor masses, supporting the role of JQ1 in modulating the IL-20RA/PD-L1 axis in vivo (Figure 4d,e).

## 3. Discussion

Limited therapeutic options are currently available for treatment of TNBC due to its molecular heterogeneity and lack of therapeutically targetable, high-frequency driver alterations [32]. Therefore, the identification of novel molecular targets remains an ongoing challenge. In the search for alternative/additional targets, attention has focused on TME. The dynamic crosstalk between TME and TNBC cells plays a critical role in tumor progression and treatment response, representing an attractive target for developing new drugs with anticancer activity [25,33]. Among the secreted factors, cytokines present in the TME influence cell fate and drug efficacy, affecting therapeutic outcomes. Notably, IL-20 subfamily cytokines are involved in key cancer hallmarks: proliferation, resistance to cell death, angiogenesis, migration, invasion, and development of metastases [27,28]. Upon binding to their respective receptor complexes, all IL-20 subfamily members activate JAK-STAT pathway, and primarily STAT3, by supporting tumor progression [34]. Previous studies have documented a significant upregulation of IL-20RA expression across multiple malignancies, including pancreatic, colorectal, lung, and breast cancers. Furthermore, its downstream signaling pathways are actively implicated in driving key oncogenic processes, such as tumor cell proliferation, angiogenesis, and EMT-mediated metastasis [27,30,35,36,37].

Epigenetic therapies are receiving much attention in the management of various malignancies, including TNBC [38,39]. In this context, attention has focused on JQ1, a small molecule inhibitor of BET proteins which, by regulating the transcription of genes downstream of BRD4 with a high impact on the oncogenic pathways, elicits antitumor effects [17]. Consistently, BRD4 dysregulation has been strongly linked to TNBC pathogenesis, influencing tumor progression, metastatic potential, and prognosis [31,40]. The therapeutic potential of JQ1 is further underscored by its synergistic effects when combined with chemo- and immunotherapies [15,41]. Specifically, its co-administration with the HDAC inhibitor mocetinostat downregulates the RAS/MAPK pathway, inhibiting cell proliferation across ER-positive and TNBC subtypes [42]. Similarly, a triple regimen combining JQ1, paclitaxel, and an anti-PD-L1 monoclonal antibody has been reported to suppress tumor progression and extend survival in two different TNBC mouse models [43]. However, despite its remarkable efficacy in preclinical settings and combination regimens, the clinical translation of JQ1 is strictly precluded by its unfavorable drug-like properties. JQ1 exhibits poor pharmacokinetic parameters, including a very short biological half-life, and notable side effects, especially dose-limiting thrombocytopenia, gastrointestinal toxicity, and lymphoid suppression [15,21,44]. To mitigate these challenges, ongoing research is directed toward developing next-generation BET inhibitors with enhanced selectivity and improved tolerability profiles [44]. Furthermore, the application of innovative drug delivery systems and PROTAC degradation technology aims to direct the treatment specifically to tumor sites, thereby minimizing adverse systemic effects [22]. Consistent with the need for innovative delivery systems, we recently showed that the encapsulation of JQ1 in nanoparticles improved its anticancer efficacy against TNBC in vitro and in vivo by blocking cell proliferation and potentially overcoming its pharmacokinetic limitations [31,45].

Since TME critically regulates the therapeutic response of BC cells [28,46], elucidating the iBET–TME interplay could provide novel insights and perspectives in TNBC research. In this regard, Yu et al. 2021 found that the expression of IL-20RA in colon cancer cells was significantly downregulated after treatment with JQ-1 [30] and a recent evidence suggests that IL-6 modulates JQ1-mediated apoptosis in TNBC cells, supporting JQ1 as a promising therapeutic option for managing TNBC tumors characterized by an IL-6-enriched TME [46]. In the present study, we specifically investigated the downregulation of IL-20RA expression through the administration of JQ1. We first examined the expression of IL-20RA in a panel of human BC cell lines, observing consistently high levels across all lines, with the highest expression in MDA-MB-231 and MDA-MB-468 TNBC cells. In both cell lines, 24 h JQ1 exposure dose-dependently downregulated IL-20RA mRNA and protein expression and reduced phospho-STAT3 levels, suggesting a potential involvement of the canonical IL-20RA signaling pathway, which modulates tumor cell proliferation. Recent studies have identified STAT3 as a critical oncogenic driver in TNBC pathogenesis [47,48]. By activating the JAK1-STAT3-SOX2 pathway, IL-20RA upregulates the expression of PD-L1 [7,27]. While PD-L1 is traditionally recognized for driving adaptive immunosuppression in the TME by inactivating T cells, emerging evidence highlights a critical, tumor-intrinsic function of this protein that operates independently of host immunity. Specifically, tumor-intrinsic PD-L1 signaling actively promotes cancer cell survival, cancer stemness, and resistance to chemotherapeutic agents by modulating intracellular oncogenic pathways [49,50]. In the current study, we explored the influence of JQ1 on PD-L1 expression. Consistent with previous reports [7,51], we found elevated PD-L1 levels in TNBC cells which were significantly downregulated by JQ1 treatment, suggesting that this compound may modulate PD-L1 expression, at least in part, through the inhibition of the IL-20RA axis. Our data prompted us to evaluate the effects of JQ1 on IL-20RA expression in vivo, employing the MDA-MB-231 xenograft model previously established in our laboratory [31]. Treatment with JQ1 (20 mg/kg) suppressed IL-20RA expression and affected the JAK/STAT signaling pathway, as evidenced by decreased phospho-STAT3 levels, compared to untreated animals. Additionally, downregulation of PD-L1 expression was observed in the tumor masses, suggesting that JQ1 impairs a tumor-intrinsic survival and chemo-resistance mechanism driven by PD-L1 rather than active immune evasion. It is worth noting that while our findings establish a clear association between JQ1 treatment and the suppression of the IL-20RA/p-STAT3 axis, they demonstrate a correlation rather than a definitive causal dependence of the JQ1-induced phenotype on this pathway. Future genetic rescue or overexpression studies will be required to confirm whether the therapeutic efficacy of JQ1 is strictly dependent on IL-20RA signaling. Furthermore, because the nude mouse xenograft model used in this study lacks mature T cells, it restricts our observations regarding PD-L1 to T-cell-independent mechanisms. Future studies utilizing syngeneic mouse models or humanized mice are necessary to fully elucidate the impact of this pathway on T-cell-mediated anti-tumor immunity.

In the present work, we characterized the unexplored interplay between JQ1 and IL-20RA in TNBC. Our findings identified IL-20RA as a novel downstream target modulated by JQ1 and further investigations are required to support its employment as a viable therapeutic option for tumors exhibiting high IL-20RA expression.

## 4. Materials and Methods

### 4.1. Cell Culture

Human breast epithelial cell lines MCF-10A and human BC cell lines MDA-MB-157, MDA-MB-231, MDA-MB-468 and MCF-7 were purchased from the American Type Culture Collection (Manassas, VI, USA). These cell lines were authenticated by short-tandem repeat profiling. MDA-MB-157, MDA-MB-231, MDA-MB-468 and MCF-7 were cultured in DMEM containing FBS 10% and penicillin/streptomycin 1%. MCF-10A were cultured in DMEM/Ham′s Nutrient Mixture F-12 supplemented with horse serum 5%, cholera toxin 1 ng/mL, human insulin 10 µg/mL, epidermal growth factor 20 ng/mL and hydrocortisone 0.5 µg/mL. All cells were maintained in an incubator at 37 °C with 5% CO_2_.

### 4.2. In Vivo Experiments

In vivo experiments were conducted as part of our previously published study [31]. In the present work, we utilized archived tumor tissues from that experimentation. Briefly, five-week-old female athymic nude mice were subcutaneously injected with MDA-MB-231 cells, and treatment was initiated when tumors reached approximately 150 mm^3^. JQ1 (Selleck Chemicals) was diluted in PBS containing 5% PEG400 and 5% Tween 80. Mice received intraperitoneal (i.p.) injections of JQ1 (20 mg/kg), 5 days a week for two weeks. At the end of the treatments, animals were anesthetized with Avertin (2,2,2-Tribromoethanol, 250 mg/kg, i.p.) and euthanized by cervical dislocation. Tumor tissues were harvested and weighed; a portion of each tumor was immediately snap-frozen in liquid nitrogen and stored at −80 °C, while the remaining part was fixed in 10%. In the present study, we used frozen tumor tissues derived from both the vehicle-treated group and the JQ1-treated group for downstream gene and protein expression analyses.

### 4.3. Extraction of RNA and Gene Expression Studies

Total RNA extraction, quantification, reverse transcription, and quantitative real-time PCR (qPCR) were performed using reagents, kits, and instrumentation entirely sourced from Thermo Fisher Scientific Inc. (Waltham, MA, USA). Briefly, RNA extraction from both BC cells and tumor tissues was performed using TRIzol reagent (Thermo Fisher Scientific Inc., Waltham, MA, USA) in compliance with the manufacturer’s instructions [52]. A NanoDrop Spectrophotometer (Thermo Fisher Scientific Inc., Waltham, MA, USA) was employed to determine the concentration and purity of the isolated RNA. Subsequently, reverse transcription was carried out utilizing the High-Capacity cDNA Reverse Transcription kit (Thermo Fisher Scientific Inc., Waltham, MA, USA). Quantitative real-time PCR (qPCR) was then conducted on a QUANT STUDIO 3 instrument using the SYBR™ Green qPCR Master Mix (Thermo Fisher Scientific Inc., Waltham, MA, USA). Data analysis and quantification were managed via the QUANT STUDIO REAL-TIME PCR Software (v1.4.3). Final results were determined by the comparative 2^−∆∆Ct^ method using GAPDH as an endogenous control. Data were expressed as mean ± S.D. of three replicates. The following primers were used: human IL-20RA gene, 5′-CCAGAGGGTCTTCAAGGAGTTAA-3′ and 3′-GGCATAATACTGGTGTTCGTAGT-5′; human GAPDH gene, 5′-CCAAGGAGTAAGACCCCTGG-3′ and 3′-TGGTTGAGCACAGGGTACTT-5′. After all analysis, expression levels in tumors of the control groups were averaged and indicated as 1, and the expression levels in tumors treated with JQ1 were expressed as the ratio to the average levels of tumors in the control groups.

### 4.4. Protein Extraction and Western Blotting

Total proteins were extracted from cells by pre-heated 1% SDS solution as previously described [36] and tumor tissue, homogenizing the samples in modified ELB (Erythrocyte Lysis Buffer) buffer 50 mM Hepes pH 7, 250 mM NaCl, 0.5% NP40, 1 mM PMSF (Phenylmethylsulfonyl fluoride), 10 mM sodium pyrophosphate, 1 mM sodium orthovanadate, 1 mM sodium fluorure, 1 mM DTT (Dithiothreitol) and protease inhibitor cocktail as previously described [53]. In total, 30 μg of total protein were run on a 9 or 12% SDS-PAGE gel, transferred to PVDF membrane, blocked with PBS, 0.1% Triton, and 5% non-fat dry milk (T-PBS) and incubated overnight with affinity-purified anti-IL-20RA (PA1-41167; PA5-119916, Thermo Fisher Scientific Inc.), anti-JAK, anti-phospho JAK, anti-STAT3, anti-phospho STAT3, anti-PD-L1 (Cell Signaling Technology, Beverly, MA, USA) anti-β-actin and anti-GAPDH antibodies (Merk Life Sciences, Milan, Italy), diluted 1:1000, 1:1000, 1:1000, 1:1000, 1:500, 1:1000, 1:5000 and 1:5000, respectively. Then, the membranes were incubated with horseradish peroxidase-conjugated anti-rabbit or anti-mouse antibody (BD Biosciences, Milan, Italy) and the proteins were visualized by using a chemiluminescence system (ECL Plus, Revvity, Milan, Italy).

### 4.5. Statistical Analysis

All results are expressed as mean ± standard deviation (SD) and were considered statistically significant if *p*-values were lower than 0.05. Results were analyzed by one-way ANOVA followed by the Tukey–Kramer multiple comparisons test or T-test with Welch’s correction. All statistical analyses were performed using GraphPad Prism version 9.3.0 statistical software (GraphPad Software Inc., San Diego, CA, USA).

## Data Availability

The original contributions presented in this study are included in the article. Further inquiries can be directed to the corresponding author.
